# Phylogenetic analysis of strains of *Orf virus *isolated from two outbreaks of the disease in sheep in Greece

**DOI:** 10.1186/1743-422X-9-24

**Published:** 2012-01-19

**Authors:** Charalambos Billinis, Vasia S Mavrogianni, Vasiliki Spyrou, George C Fthenakis

**Affiliations:** 1 Veterinary Faculty, University of Thessaly, Karditsa, Greece; 2 Department of Animal Production, Technological Educational Institute of Larissa, Larissa, Greece

**Keywords:** Contagious ecthyma, Genital orf, Greece, Mastitis, Orf, Phylogenetic analysis, Sheep, Teat

## Abstract

**Background:**

Although orf is endemic around the world, there are few descriptions of *Orf virus *strains and comparisons of these strains. We report the sequence and phylogenetic analysis of the partial B2L gene of *Orf virus *from two outbreaks of the disease in Greece. The first was an outbreak of genital form of the disease in a flock imported from France, whilst the second was an outbreak of the disease in the udder skin of ewes and around the mouth of lambs in an indigenous flock.

**Results:**

Phylogenetic analysis was performed on a part (498 bp) of the B2L gene of 35 *Parapoxvirus *isolates, including the two *Orf virus *isolates recovered from each of the two outbreaks in the present study. This analysis revealed that the maximum nucleotide and amino-acid variation amongst *Orf virus *strains worldwide (n = 33) was 8.1% and 9.6%, respectively. The homology of the nucleotide and amino-acid sequences between the two Greek isolates was 99.0% and 98.8%, respectively. The two Greek isolates clustered only with *Orf virus *strains.

**Conclusions:**

We suggest that there can be differences between strains based on their geographical origin. However, differences in the origin of strains or in the clinical presentation of the disease may not be associated with their pathogenicity. More work is required to determine if differing clinical presentations are linked to viral strain differences or if other factors, e.g., flock immunity, method of exposure or genetic susceptibility, are more important to determine the clinical presentation of the infection.

## Background

Contagious echtyma ('orf') is a contagious disease, caused by the epitheliotropic *Orf virus*, a member of the genus *Parapoxvirus*. The disease has a worldwide distribution and a significant financial importance. The disease affects primarily sheep and goats; camels, South American camelids, Cervidae (deer, reindeer), other ruminants (bighorn sheep, chamois, dall sheep, mountain goats, musk oxen, serows, steenboks, tahr), dogs, cats and squirrels. The disease also has a zoonotic potential, although it is more of an occupational hazard to people working with animals (e.g., farmers, animal carers, veterinarians).

Clinical features of the infection vary. In some animals, infection may remain subclinical; however, occasionally and especially in young animals, case fatality may reach up to 80% [1]. In lambs, lesions are usually localised around the mouth and the nostrils, frequently originating at the commissures of the lips; lesions can also be seen within the buccal cavity (gums, hard palate, tongue) and, occasionally, in the oesophagus or the abomasum. In ewes, lesions are primarily observed on the teat (usually, around the teat orifice) or the udder skin and less often in the inguinal area and the thigh [2]. In adult animals, lesions of the disease can also be found in the genital organs (ewes: vulva and skin-vaginal junction, rams: preputial orifice) [3], as well as in the coronet [4]. Lesions follow a well-defined development pattern: local erythema, followed by formation of papules, vesicles, postules and scabs. As lesions resolve, scabs become dry and are shed, with no scar remaining at the lesion site.

The virus genome includes linear double-stranded DNA. The envelope gene (B2L) of the virus encodes a highly immunogenic major envelope protein of molecular weight about 42 kDa [5]. This gene has been widely used for molecular characterisation and phylogenetic analysis of strains of the virus [6-9].

Although the disease is endemic around the world, there are few descriptions of *Orf virus *strains and comparisons of these strains between them. Details of 31 *Orf virus *strains, whose sequence of the envelope gene (B2L) has been reported before and which were used in the present study, are presented in Table 1. Of these strains, only five (one each from sheep, goats, reindeer, chamois and mountain goats) had been isolated in Europe. All strains had been isolated from skin lesion form of the disease.

**Table 1:** Information about *Parapoxvirus *strains (n = 35) used for phylogenetic analysis

No	Strain identification	Animal species infected	Country of origin	**GenBank accession no**.	Reference
1	Cro-Cres-12446/09	Sheep	Croatia	HQ215589	[10]

2	Vaccine strain	Goat	USA	AY278209	[11]

3	Ena	Serow	Japan	AB521175	[6]

4	NZ2/2004	Sheep	New Zealand	AY453667	[12]

5	His	Sheep	Japan	AB189670	[13]

6	MTO5	Sheep	Brazil	FJ665818	[7]

7	Hoping	Goat	Taiwan	EU935106	[14]

8	Cro-Goat-11727/10	Goat	Croatia	HQ215588	[10]

9	Hub/2009	Goat	China	GU320351	[9]

10	GRE-1 genital 2003	Sheep	Greece	JN368482	Present study

11	GRE-2 teat 2004	Sheep	Greece	JN368483	Present study

12	NE1	Goat	Brazil	FJ665819	[7]

13	Taiping	Goat	Taiwan	EU327506	[14]

14	ORFV/2009/Korea	Goat	Korea	GQ328006	[8]

15	[*unidentified*]	[*unknown*]	Iran	AY958203*	[*Ghorashi et al. (unpublished)*]

16	Jilin	Sheep	China	FJ808074	[15]

17	India 67/04	Sheep	India	DQ263305	[16]

18	*Pseudocowpox virus *strain	Cattle	-	AY424972	[11]

19	F00.120R	Reindeer	Finland	AY453656	[12]

20	*Bovine papular stomatitis virus *strain	Cattle	-	AY424973	[11]

21	Assam09	Goat	India	JN846834*	[*Bora et al. (unpublished)*]

22	ORFV/LiaoNing/2010/China	Goat	China	HQ694773*	[*Zhang et al. (unpublished)*]

23	ORFV/GanSu/2009/China	Sheep	China	HQ694772*	[*Zhang et al. (unpublished)*]

24	Isolate A	Goat	Brasil	JN088053*	[*Abrahao et al. (unpublished)*]

25	Isolate D	Sheep	Brasil	JN088052*	[*Abrahao et al. (unpublished)*]

26	Isolate A	Goat	Brasil	JN088051*	[*Abrahao et al. (unpublished)*]

27	Shanxi	Goat	China	HQ202153*	[*Shi (unpublished)*]

28	JS04	Sheep	China	GU903501	[17]

29	Cam/09	Camel	India	GU460370*	[*Venkatesan et al. (unpublished)*]

30	Muk/2000	Goat	India	HM466933*	[*Venkatesan et al. (unpublished)*]

31	GE	Serow	Japan	AB493826	[18]

32	Asahi	Serow	Japan	AB521170	[19]

33	485/09	Chamois	Italy	HQ239073*	[*Scagliarini et al. (unpublished)*]

34	378/08	Mountain goat	Italy	HQ239072*	[*Scagliarini et al. (unpublished)*]

35	NZ-2/1994	Sheep	New Zealand	OVU06671	[5]

Strains 1-17, 19 & 21-35: *Orf virus*, *: information regarding this strain drawn through GenBank accession no.

In this paper, we report the sequence and phylogenetic analysis of the B2L gene of *Orf virus *from two outbreaks of the disease in Greece. The first was an outbreak of the genital form of the disease in a flock imported from France, whilst the second was an outbreak of the disease in the udder skin of ewes and around the mouth of lambs in an indigenous flock. This is the first information comparing *Orf virus *strains isolated in Greece and their relationship with strains isolated in other parts of the world based on B2L gene. This is also the first information regarding an *Orf virus *strain isolated from the genital form of the disease.

## Case presentation

**Figure 1:**
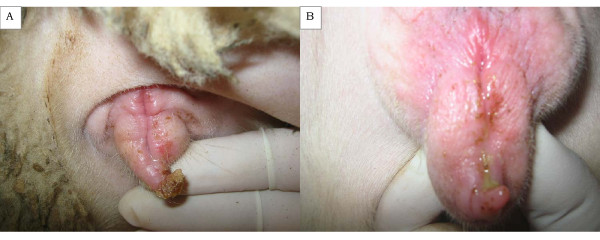
**Representative lesions of genital form of orf in ewes**. (**a**) Orf scabs scattered in the lower part of the vulva and the entrance of the vagina of a ewe. (**b**) Purulent vaginal discharge in a ewe with genital form of orf.

The first outbreak occurred in 2003 and involved cases of genital form of orf. It was recorded in an intensive dairy flock with 115 Lacaune-breed ewes, in the region of Peloponnese in South Greece (coordinates: 37.06 °N, 21.63 °E). Animals in the flock were imported from France after the end of the breeding season. Cases were diagnosed 2 months after the end of the mating season, soon after establishment of the animals in the farm. Clinically, lesions characteristic of orf (papules, postules and scabs) were recorded in 78 ewes. Lesions were localised in the lower part of the vulva and the entrance of the vagina (Figure 1a); their removal was followed by mild bleeding. In 10 ewes, purulent vaginal discharge was evident (Figure 1b). The lesions progressively resolved and, subsequently, all ewes lambed normally. After lambing, cases of orf were detected in the teats of 24 ewes of those previously affected, as well as around the lips and the nostrils of their lambs. No cases of the disease were recorded in the farm staff.

**Figure 2:**
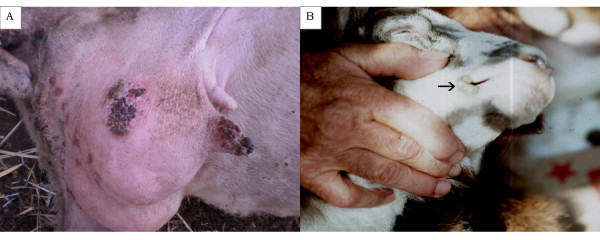
**Representative lesions of orf in the teats of ewes and around the lips and the nostrils of their lambs**. (**a**) Orf scabs in the body of a teat and the skin of the udder of a ewe. (**b**) Orf lesions in the commissures of the lips of a lamb.

The second outbreak occurred in 2004 and involved cases of orf in the teats of ewes and around the lips and the nostrils of their lambs in an indigenous semi-intensive dairy flock with 220 Karagouniko-breed ewes, in the region of Thessaly in Central Greece (coordinates: 39.39 °N, 21.94 °E). The famer reported that outbreaks of the disease had been occurring in the flock for some years. Clinical cases with lesions characteristic of orf (papules, postules and scabs) were recorded *post-partum *in 18 lactating ewes and in their lambs. In ewes, lesions were localised in the body of the teats and around the teat orifice (Figure 2a); in lambs, lesions were localised in the corners of the lips (Figure. 2b). All cases, in ewes and lambs, recovered spontaneously. No cases of the disease were recorded in the farm staff.

In both outbreaks, a tentative clinical diagnosis of orf was made on clinical evidence. Material was collected and submitted for virological examination from 25 animals (genital system and teats) in the first farm and from all affected ewes (n = 18; teats) and 10 lambs (lips) in the second farm. In each case, scabs formed over the lesions (2-3 scabs from each animal) were collected and transferred to the laboratory within 24 h; 10% suspension in PBS, pH 7.2 was prepared.

DNA was isolated from the suspension, by using a commercial kit (Gentra Systems, Minneapolis, USA), according to the manufacturer's instructions. PCR amplification was carried out according to the guidelines described by Kwok and Higuchi [20]. The sequence of the primers and the PCR conditions to amplify a part of 594 bp of B2L gene of *Orf virus*, were the same as previously described [6] with slight modifications. More specifically, the Invitrogen PCR selection kit (Invitrogen, Carlsbad, CA, USA) was used. Each reaction mixture (total volume: 25 μL) contained 5 μL 1 × buffer, 1.5 mM MgCl_2_, 1 μL deoxynucleoside triphosphate mix (final concentration of each dNTP: 0,2 mM), 0,2 μL Platinum *Taq *DNA polymerase, 100 ng of extracted DNA and 25 pmoles of the primers PPP-1 5'-gtc gtc cac gat gag gag ct-3' and PPP-4 5'-tac gtg gga agc gcc tcg ct-3' (based on the previously published sequence of the B2L gene of *Orf virus *strain NZ2/2004 [12]). Following an initial denaturation for 2 min at 94°C, the reaction mixture was subjected to 35 cycles of heat denaturation at 94°C for 35 s, primer annealing at 60°C for 35 s and DNA extension at 72°C for 45 s, completed by a final extension of 5 min at 72°C.

Following amplification, 10 μL of each PCR product was analysed by electrophoresis on 2% agarose gel and stained with ethidium bromide (0.5 mg mL^-1^). A 100 bp DNA ladder was analysed on the same gel to serve as a size marker. As negative control, DEPC-treated H_2_O was used instead of DNA in PCR assay, to exclude any contamination. The positive PCR products were purified by using the PureLink PCR Purification Kit (Invitrogen, Carlsbad, CA, USA) according to the manufacturers' instructions. Sequence analysis in both directions was performed commercially by MWG Biotech (Ebersberg, Germany). All samples were analysed twice and only high-quality sequences were used.

Extracted DNA from material submitted and examined from all animals, from both flocks was positive for *Orf virus*. From both outbreak 1 and outbreak 2, all isolates within each outbreak were identical based on the analysis of part of B2L gene. However, different isolates were recovered from each of the two outbreaks. We designated the isolates by the codes 'GRE-1 genital 2003' (recovered from the first outbreak, i.e., from cases of genital form of orf) and 'GRE-2 teat 2004' (recovered from the second outbreak, i.e., from cases of orf in the teats of ewes and around the lips and the nostrils of their lambs).

**Figure 3:**
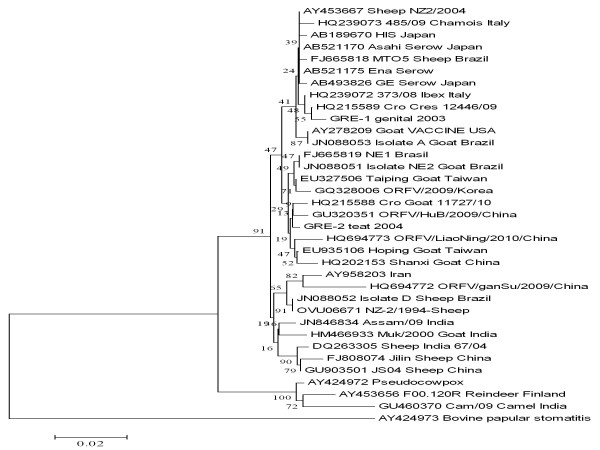
**A neighbour-joining phylogenetic tree, using Kimura-2 parameter distance matrix, from 35 (including those in present study) *Parapoxvirus *strain sequences (partial sequences of B2L gene)**. GenBank accession numbers and geographic origin of strains are shown. Bootstrap values (%) are represented at each tree node. Node support was assessed with 1,000 bootstrap pseudo-replicates.

The partial sequence of the major envelope gene of the two strains of *Orf virus*, i.e. 'GRE-1 genital 2003' and 'GRE-2 teat 2004', were submitted to GenBank with accession numbers JN368482 and JN368483, respectively. Nucleotide sequences from other *Parapoxvirus *isolates were retrieved from Genbank (NCBI). Phylogenetic and molecular evolutionary analyses were conducted using program MEGA 3.1 [21]. A neighbour-joining phylogenetic tree using Kimura-2 parameter distance matrix [22] was inferred from sequence of 35 *Parapoxvirus *strains; these included a *Bovine papular stomatitis virus *strain, *Pseudocowpox virus *strain, 31 *Orf virus *strains previously isolated by other researchers and the two strains isolated in the present study (Figure. 3). Node support was assessed with 1,000 bootstrap pseudo-replicates. Detailed information of *Parapoxvirus *strains analysed is in Table 1.

Phylogenetic analysis was performed on a part (498 bp) of B2L gene 35 *Parapoxvirus *strains, including the two strains recovered in the present study. This analysis revealed that the maximum nucleotide and amino-acid variation amongst *Orf virus *strains isolates worldwide (n = 33) was 8.1% and 9.6%, respectively. The homology of the nucleotide and amino-acid sequences between the two Greek isolates was 99.0% and 98.8%, respectively. The two Greek isolates clustered only with *Orf virus *strains (Figure 3).

The 'GRE-1 genital 2003' *Orf virus *strain (isolated from cases of genital form of orf) clustered together with the following strains: Cro-Cres 12446/09 from Croatia, Vaccine strain from USA, Ena, HIS, GE and Asahi from Japan from Japan, NZ2/2004 from New Zealand, MTO5 and Isolate A from Brazil, and 485/09 and 373/08 from Italy. It shared the highest homology with Cro Cres 12446/09 (99.6% homology) at nucleotide level and with Cro-Cres 12446/09, Ena, His, GE, Asahi, NZ2/2004, MTO5, 485/09 and 373/08 (100% homology) at amino-acid level.

The 'GRE-2 teat 2004' *Orf virus *strain (isolated from cases of orf in the teats of ewes and around the lips and the nostrils of their lambs) clustered together with the following strains: Hoping and Taiping from Taiwan, Cro-Goat 11727/10 from Croatia, Hub/2009 and ORFV/LiaoNing/2010/China from China, NE1 from Brazil and ORFV/2009/Korea from Korea. It shared the highest homology with NE1 (99.6% homology) at nucleotide level and with Hoping, Cro-Goat 11727/10, Hub/2009 and Shanxi (100% homology) at amino-acid level.

**Figure 4:**
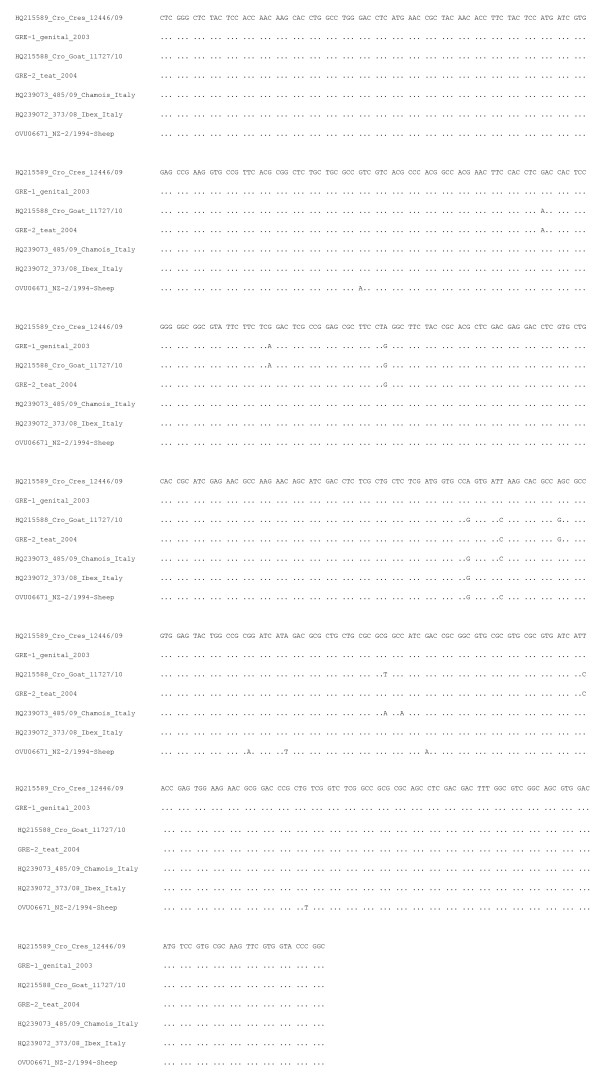
**Alignment of 498 nucleotide sequences of the B2L gene from 7 (including those in the present study) *Orf Virus *strains**. Identical nucleotides within the sequence are marked by dots.

The percent diversity of the nucleotide sequence of the partial B2L gene between the 33 *Orf virus *strains is shown in Table 2, whilst alignment of nucleotide sequences of the B2L gene from 7 (including those in the present study) *Orf Virus *strains is shown in Figure. 4.

**Table 2:** Percent nucleotide sequence diversity of the partial B2L gene between 35 *Parapoxvirus *strains

	**Strain no**.																																		
**Strain no.-GenBank accession no**.	**1**	**2**	**3**	**4**	**5**	**6**	**7**	**8**	**9**	**10**	**11**	**12**	**13**	**14**	**15**	**16**	**17**	**18**	**19**	**20**	**21**	**22**	**23**	**24**	**25**	**26**	**27**	**28**	**29**	**30**	**31**	**32**	**33**	**34**	**35**

01-HQ215589																																			

02-JN368482	0.4																																		

03-HQ215588	1.6	1.2																																	

04-FJ808074	2.7	3.1	2.7																																

05-DQ263305	2.3	2.7	2.3	1																															

06-AY424973	18.3	18.3	18.3	19.1	18.6																														

07-AY424972	5.4	5.4	5	4.6	3.9	18.3																													

08-EU935106	1.2	1.6	0.8	2.3	1.8	18.1	5																												

09-GU320351	1.6	1.6	0.8	2.7	2.3	18.3	5	0.8																											

10-FJ665819	1.4	1.4	0.6	2	1.6	17.6	4.3	0.6	0.6																										

11-EU327506	1.2	1.2	0.8	2.3	1.8	17.6	4.6	0.8	0.8	0.2																									

12-FJ665818	0.6	1	1.4	2.5	2	18.3	5.2	1	1.4	1.2	1																								

13-AY278209	0.8	1.2	1.6	2.2	1.8	18.3	5	1.2	1.6	1.4	1.2	0.6																							

14-JN368483	1	1	0.6	2.5	2	17.8	4.8	0.6	0.6	0.4	0.6	1.2	1.4																						

15-GQ328006	1.6	1.6	1.2	2.7	2.2	17.8	5	1.2	1.2	0.6	0.4	1.4	1.6	1																					

16-AY958203	2.5	2.9	2.9	2.5	1.8	19.1	4.6	2.5	2.9	2.3	2	2.3	1.8	2.7	2.5																				

17-AY453656	6.3	6.3	5.9	5.4	4.8	19.1	1.2	5.9	5.9	5.2	5.4	6.1	5.9	5.6	5.8	5.4																			

18-AY453667	0.4	0.8	1.2	2.3	1.8	18.1	5	0.8	1.2	1	0.8	0.2	0.4	1	1.2	2	5.9																		

19-AB521175	0.4	0.8	1.2	2.3	1.8	18.1	5	0.8	1.2	1	0.8	0.2	0.4	1	1.2	2	5.9	0																	

20-AB189670	0.6	1	1.4	2.5	2	18.3	5.2	1	1.4	1.2	1	0.4	0.6	1.2	1.4	2.3	6.1	0.2	0.2																

21-JN846834	1.8	1,8	1.8	1.6	1.4	18.3	4.1	1.8	1.8	1.6	1.4	1.6	1.4	1.6	1.8	2	5	1.4	1.4	1.6															

22-HQ694773	2	2	1.2	3.1	2.7	18.1	5.4	0.8	1.2	1	1.2	1.8	2	1	1.6	3.3	6.3	1.6	1.6	1.8	2.3														

23-HQ694772	3.7	4.2	4.2	3.5	3.3	20.7	6.1	3.7	4.2	3.5	3.3	3.1	3.3	3.9	3.7	2.3	7	3.3	3.3	3.5	2.7	4.4													

24-JN088053	0.8	1.2	1.6	2.2	1.8	18.3	5	1.2	1.6	1.4	1.2	0.6	0	1.4	1.6	1.8	5.9	0.4	0.4	0.6	1.4	2	3.3												

25-JN088052	1.4	1.8	2.2	1.6	1.4	18.6	4.3	1.8	2.2	1.6	1.4	1.2	1	2	1.8	1.2	5.2	1	1	1.2	1.2	2.7	2.3	1											

26-JN088051	1.4	1.4	0.6	2	1.6	17.6	4.3	0.6	0.6	0	0.2	1.2	1.4	0.4	0.6	2.3	5.2	1	1	1.2	1.6	1	3.5	1.4	1.6										

27-HQ202153	1.4	1.6	1.2	2.9	2.5	18.9	5.6	0.6	1.4	1.2	1.4	1.6	1.8	1.2	1.8	3.1	6.5	1.4	1.4	1.6	2.5	1.4	4.4	1.8	2.5	1.2									

28-GU903501	2	2.5	2	0.6	0.4	18.6	3.9	1.6	2	1.4	1.6	1.8	1.6	1.8	2	1.8	4.8	1.6	1.6	1.8	1	2.5	2.9	1.6	1	1.4	2.2								

29-GU460370	7.4	7.4	6.9	6.5	5.8	20.2	2.4	7	6.9	6.3	6.5	7.2	6.9	6.7	6.9	6.5	2.9	7	7	7.2	6.1	7.4	8.1	6.9	6.3	6.3	7.6	5.8							

30-HM466933	2.3	2.3	1.8	2	1.6	19.1	4.3	1.8	1.8	1.6	1.8	2	1.8	1.6	2.3	2.7	5.2	1.8	1.8	2	1.2	2.3	3.9	1.8	2	1.6	2.5	1.4	6.3						

31-AB493826	0.6	1	1.4	2.5	2	18.3	5.2	1	1	1.2	1	0.4	0.6	1.2	1.4	2.2	6.1	0.2	0.2	0.4	1.6	1.8	3.5	0.6	1.2	1.2	1.6	1.8	7.2	2					

32-AB521170	0.4	0.8	1.2	2.3	1.8	18.1	5	0.8	1.2	1	0.8	0.2	0.4	1	1.2	2	5.9	0	0	0.2	1.4	1.6	3.3	0.4	1	1	1.4	1.6	7	1.8	0.2				

33-HQ239073	0.8	1.2	1.4	2.7	2.3	18.6	5.4	1.2	1.6	1.4	1.2	0.6	0.8	1.4	1.6	2.5	6.3	0.4	0.4	0.6	1.8	2	3.7	0.8	1.4	1.4	1.8	2	7.4	2.3	0.6	0.4			

34-HQ239072	0.2	0.6	1.4	2.5	2	18.3	5.2	1	1.4	1.2	1	0.4	0.6	1.2	1.4	2.3	6.1	0.2	0.2	0.4	1.6	1.8	3.5	0.6	1.2	1.2	1.2	1.8	7.2	2	0.4	0.2	0.6		

35-OVU06671	1.4	1.8	2.2	1.6	1.4	18.6	4.3	1.8	2.2	1.6	1.4	1.2	1	2	1.8	1.2	5.2	1	1	1.2	1.2	2.7	2.3	1	0	1.6	2.5	1	6.3	2	1.2	1	1.4	1.2	

Strains 1-5 & 8-35: *Orf virus*, strain 6: *Bovine papular stomatitis virus*, strain 7: *Pseudocowpox virus*

## Discussion

There are few reports of characterisation of *Orf virus *strains by using the B2L gene, from world-wide sources, with only five of the strains described isolated in Europe prior to this study. All strains have been isolated from the udder skin of ewes or from lambs. This is the first genetic characterisation of a strain from the genital form of the disease. In a previous phylogenetic analysis of *Orf virus *strains from Greece and Italy [23], the VIR gene of the virus was amplified; hence the results are not comparable to the present ones.

Reports of the genital form of the disease in ewes are limited to the description of the skin lesions. Vaginitis (as indicated by the purulent discharge from the animals; Figure. 1b) has never been reported. However, in all cases, pregnancy was carried to term in all affected animals. In the second outbreak, animals from the flock infected with the 'GRE-2 teat 2004' strain were subsequently used in an experimental study, where it was shown that *Orf virus *infection in teats of ewes leads to depletion of local mammary defences and predisposes animals to mastitis [24]. Those experimental findings were allied to the clinical evidence recorded in the same farm, where higher frequency of mastitis was recorded among the *Orf virus*-infected animals [25].

One may suggest that there can be differences between strains based on their origin (one strain isolated from animals imported from France, the other strain isolated from indigenous animals). Unfortunately, we could not find reports in the literature with genetic characterisation of *Orf virus *strains from France, which could have been used to compare with those of strain 'GRE-1 genital 2003'. Lojkic et al. [10] have also postulated that import of animals to their country (Croatia) has led to import of *Orf virus *strains in there. The genital form of the disease indicates a venereal transmission from the rams in France, before animals were transported to Greece. As the virus can persistently infect animals, one may suggest that the imported sheep had already been subclinically infected and the coupled transportation stress - pregnancy immunosuppression led to development of clinical disease.

## Conclusions

No previous reports characterising *Orf virus *strains from the genital form of the disease exist. Hence, it will be interesting to study relationship of strains from different clinical entities of the disease. Based on current evidence, one may suggest that difference between strains did not seem to be associated with differing clinical signs, as animals with the genital form of the disease (first outbreak) subsequently developed lesions in their teats. Perhaps, differences in B2L gene may not reflect differences in clinical disease caused by the strains. Moreover, differences in the origin of strains may not be associated with their pathogenicity. In further support of that, it is noteworthy that a New Zealand strain is used in a vaccine to protect against the disease, which is licenced and widely used in European countries [1]. More work is required to determine if differing clinical presentations are linked to viral strain differences or if other factors (e.g., flock immunity, method of exposure or genetic susceptibility) are more important to determine the clinical presentation of the infection.

## Abbreviations

DNA: Deoxyribonucleic acid; dNTP: Deoxyribonucleotide; PBS: Hosphate-buffered saline; PCR: Polymerase chain reaction.

## Competing interests

The authors declare that they have no competing interests.

## Authors' contributions

CB: virological studies and manuscript preparation; VSM: clinical work; VS: virological studies; GCF: clinical work and manuscript preparation. All authors read and approved the final manuscript.
